# X-ray dark-field radiography facilitates the diagnosis of pulmonary fibrosis in a mouse model

**DOI:** 10.1038/s41598-017-00475-3

**Published:** 2017-03-23

**Authors:** Katharina Hellbach, Andre Yaroshenko, Konstantin Willer, Thomas M. Conlon, Margarita B. Braunagel, Sigrid Auweter, Ali Ö. Yildirim, Oliver Eickelberg, Franz Pfeiffer, Maximilian F. Reiser, Felix G. Meinel

**Affiliations:** 1 Institute of Clinical Radiology, Ludwig-Maximilians-University Hospital Munich, Munich, Germany; 2 Lehrstuhl für Biomedizinische Physik, Physik-Department & Institut für Medizintechnik, Technische Universität München, Garching, Germany; 3 Comprehensive Pneumology Center, Institute of Lung Biology and Disease, Helmholtz Zentrum Munich and Ludwig-Maximilians-University Hospital Munich, Munich, Germany; 4 German Center for Lung Research (DZL), Munich, Germany

## Abstract

The aim of this study was to evaluate whether diagnosing pulmonary fibrosis with projection radiography can be improved by using X-ray dark-field radiograms. Pulmonary X-ray transmission and dark-field images of C57Bl/6N mice, either treated with bleomycin to induce pulmonary fibrosis or PBS to serve as controls, were acquired with a prototype grating-based small-animal scanner. Two blinded readers, both experienced radiologists and familiar with dark-field imaging, had to assess dark-field and transmission images for the absence or presence of fibrosis. Furthermore readers were asked to grade their stage of diagnostic confidence. Histological evaluation of the lungs served as the standard of reference in this study. Both readers showed a notably higher diagnostic confidence when analyzing the dark-field radiographs (p < 0.001). Diagnostic accuracy improved significantly when evaluating the lungs in dark-field images alone (p = 0.02) or in combination with transmission images (p = 0.01) compared to sole analysis of absorption images. Interreader agreement improved from good when assessing only transmission images to excellent when analyzing dark-field images alone or in combination with transmission images. Adding dark-field images to conventional transmission images in a murine model of pulmonary fibrosis leads to an improved diagnosis of this disease on chest radiographs.

## Introduction

In pulmonary fibrosis, healthy lung tissue is destroyed and replaced by connective tissue^1,2^. The diagnosis of fibrosis remains challenging using conventional X-ray imaging, especially in early and moderate stages of the disease: As the density of air-filled lung tissue is rather small, mainly severe changes in tissue structure due to scarring and atelectasis, as they appear in pulmonary fibrosis, might lead to a visible increase of absorption signal intensity in conventional transmission images^3–5^.

X-ray dark-field radiography visualizes the small-angle scattering of X-rays at tissue-air interfaces^6–8^. As healthy pulmonary tissue yields an exceedingly strong dark-field signal, even small changes in lung structure, for example due to emphysema, lead to a significant decrease in signal strength^9,10^. As a result this may make dark-field imaging more convenient for the detection of pulmonary diseases compared to transmission radiography^11^.

Dark-field images are acquired by introduction of a three grating Talbot-Lau interferometer into the X-ray beam, where one grating is placed between the X-ray source and the sample and another two gratings are mounted between the sample and the detector. Images are gained while moving one of the gratings perpendicularly to the beam propagation direction over one grating period. Conventional transmission, differential phase-contrast and dark-field images are obtained from the recorded pictures by applying Fourier analysis^12–14^.

Since so far the diagnostic value of dark field imaging was evaluated for pulmonary emphysema^11^, the aim of the presented reader study was to investigate whether this technology offers incremental diagnostic value for diagnosing fibrosis *in vivo* compared to conventional X-ray imaging. The results of this reader study emphasize the superiority of dark field over transmission imaging as diagnostic accuracy, interreader agreement as well as diagnostic confidence significantly increase when assessing dark-field images.

## Results

### Quantitative Histology

Since histology showed that the lungs of 7 animals which had received bleomycin did not show any signs of fibrosis, these mice were considered non responders and excluded from the study.

**Figure 1:**
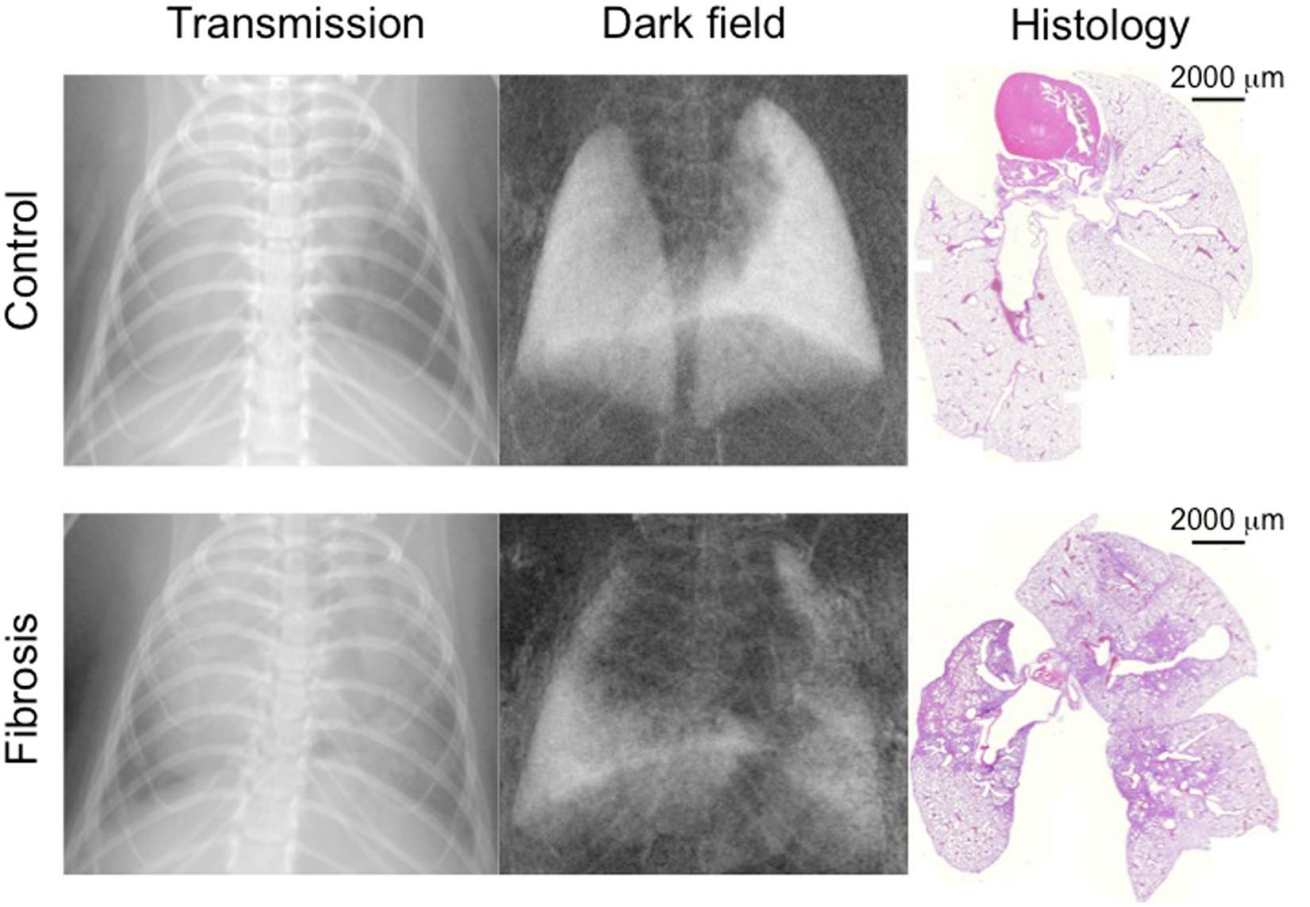
Dark-field and transmission images of fibrotic and healthy murine lungs. Representative pictures of a transmission image (left) and dark-field image (middle) of a healthy (upper row) and fibrotic lung (lower row). Histology (right) serves as the gold standard.

Tissue quantification of the remaining lungs proved that the animals in the experimental group had developed lung fibrosis. Examples of a healthy and a fibrotic lung in transmission and dark-field imaging as well as the underlying histology are depicted in Fig. 1. Tissue percentage of the lungs in the experimental group was 54.1 ± 10.5% compared to a tissue percentage of 36.0 ± 3.1% in the control group (p < 0.001).

### Quantitative Image Analysis

**Figure 2:**
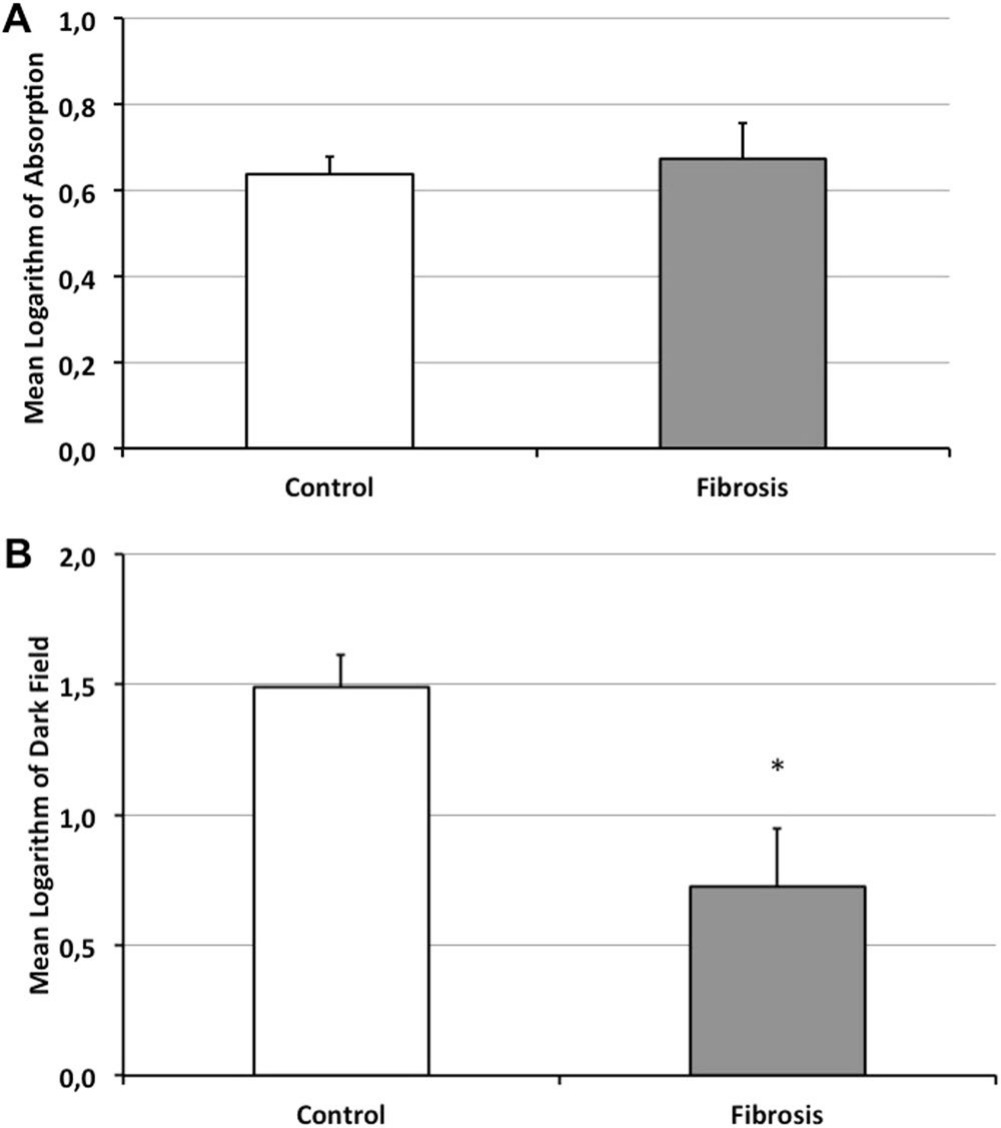
Quantitative evaluation of absorption and dark-field signal intensities. Comparison of absorption signal intensities of healthy control lungs (white column) with fibrotic lungs (grey column) did not show a statistically significant difference (**A**). In the corresponding dark-field images fibrotic lungs showed a significantly decreased dark-field signal intensity compared to healthy lungs; *p < 0.001 (**B**).

No significant change in absorption signal intensities was observed between healthy and fibrotic lungs (0.638 ± 0.04 for control lungs, 0.673 ± 0.08 for fibrotic lungs; p = 0.18) (Fig. 2A).

By contrast, mean dark-field signal intensity of the healthy controls was 1.49 ± 0.12. Lungs of the animals in the fibrosis group showed a significantly decreased signal intensity (0.725 ± 0.23; p < 0,001) (Fig. 2B).

### Reader Study

#### Diagnostic accuracy

Although both readers performed well when analyzing transmission images (area under the curve 0.9), a significant improvement in diagnostic accuracy was observed when assessing dark-field images, either alone (p = 0.02) or in combination with transmission images (p = 0.01), mainly due to a strong increase in sensitivity (Table 1).

**Table 1:** Diagnostic accuracy for separate analysis of transmission and dark-field images as well as combined readings.

Imaging modality	Sensitivity (95% Confidence Interval)	Specificity (95% Confidence Interval)	AUC	p-value*
Transmission	84.4 (67.2–94.7)	95.8 (78.9–99.9)	0.90	
Dark field	96.9 (83.8–99.9)	100.0 (85.8–100.0)	0.98	0.02
Transmission and Dark-field	100.0 (89.1–100.0)	100.0 (85.8–100.0)	1.0	0.01

AUC: Area Under the Curve. *Test for significance between transmission and dark-field readings or transmission and combined image analysis.

#### Interreader agreement

Interreader agreement for transmission images was good, with a κ value of 0.72. When analyzing dark-field images alone or in combination with transmission images Cohen’s Kappa coefficient turned out to be excellent (Kappa value 0.93 for dark-field images; Kappa value 1.00 for dark-field combined with transmission images).

#### Diagnostic confidence

Table 2 shows the mean values for diagnostic confidence indicated by both readers. Diagnostic confidences significantly increased for reader 1 (p < 0.0001) and reader 2 (p < 0.001) when analyzing dark-field images alone compared to the analysis of transmission images. Nevertheless, no further increase of diagnostic confidence could be observed when combining dark-field and transmission radiograms.

**Table 2:** Diagnostic confidence indicated by reader 1 and reader 2 for interpretation of transmission and dark-field images, either evaluated separately or combined.

Diagnostic confidence	Transmission	Dark field	Transmission and Dark field
Reader 1	1.6 ± 0.9	2.7 ± 0.5	2.7 ± 0.5
Reader 2	1.9 ± 0.8	2.7 ± 0.8	2.6 ± 0.8

## Discussion

Although numerous promising, mostly computed tomography (CT)-based imaging approaches for the detection and characterization of chronic pulmonary diseases have emerged during the last few years^15^, standard chest radiography is still the most frequently used tool for diagnostic purposes concerning the lung. Due to its wide availability as well as short scanning times and easy handling, it remains the first diagnostic step for patients presenting with symptoms of pulmonary disease^16^.

Unfortunately, due to its low sensitivity and specificity, a considerable number of diagnoses are missed in projection radiography^17^. Adding a highly sensitive new technique to this established imaging method might solve this problem.

The results of our study indicate that dark-field imaging is a valuable tool to improve the diagnosis of fibrosis on projection images compared to conventional transmission-based radiographs alone. A stronger interreader agreement and a significantly improved diagnostic confidence as well as diagnostic accuracy were found.

These results are in accordance with the outcome of a previous reader study performed on pulmonary emphysema, which found that when comparing the diagnostic value of X-ray dark-field imaging in detecting different stages of emphysema, a clear superiority over conventional transmission images could be observed^11^.

Histopathology served as the standard of reference for this study. Seven animals that had received bleomycin did not show measurable histological signs of fibrosis and therefore needed to be excluded from the study. Quantitative signal analysis of the remaining lungs’ transmission and dark-field images yielded a faintly visible, but not significant increase in transmission signal intensities and a distinct decrease in dark-field signal intensities, indicating the presence of pulmonary fibrosis. These results are in good correlation with the signal analysis performed in a recently submitted proof-of-principle paper^18^.

As has previously been shown for pulmonary emphysema^11^ and fibrosis^18^ dark-field signal intensities show an excellent correlation with quantitative histology, which is due to the dark-field signal’s high sensitivity to morphological changes of lung tissue. Because conventional transmission images are less sensitive, especially to beginning alterations in lung tissues composition, it is widely accepted that diagnosing pulmonary fibrosis without the use of CT remains challenging^19^.

Therefore, the addition of morphological information provided by the dark-field signal to the transmission radiograms’ anatomical background, seems to be an excellent diagnostic tool. Combining the information readers obtain from both imaging modalities results in best diagnostic efficiency.

Compared to conventional clinical radiography without gratings, transmission images acquired with a grating-based radiography and similar X-ray parameters will lead to a slight decrease in SNR by a factor of about 1.42 ($\surd 2$) due to the presence of the G2 absorption grating in the beam^20^. In our fibrosis data, several readers confirmed that the image quality of the transmission images is excellent.

When thinking of the potential clinical application of the technique some challenges have to be addressed: So far, only small animal models such as mice can be used to perform dark-field imaging. Before transferring this new imaging method to a clinical setup, some technical developments are necessary: With an estimated 1.4 mGy^18^ the image acquisition dose in our study is fully acceptable even to perform longitudinal studies in animals. Nonetheless, for a conventional imaging method it is still too high to be routinely used in a clinical environment. Therefore, improvement of dose efficiency is one major task that still needs to be solved. Moreover, a larger-field of view to transfer this imaging method from small-animal studies to human applications is needed. Thus larger gratings have to be developed. Finally, a switch to higher energies up to 125 kV as being used in conventional chest X-ray imaging is a necessary step as well.

To conclude, this study indicates that the combined reading of dark-field and transmission chest radiographs allows a reliable discrimination between diseased and healthy lungs in the presented murine model of pulmonary fibrosis.

## Methods

### Small animal protocol

Permission from the Institutional Animal Care and Use Committee was obtained before animal experiments, which followed national (Gesellschaft für Versuchstierkunde/Society of Laboratory Animals (GV-SOLAS)) and international (Federation of European Laboratory Animal Science Associations (FELASA)) animal welfare guidelines.

Pulmonary fibrosis was induced by instillation of 80 μl bleomycin (Sigma-Aldrich, Munich, Germany) dissolved in sterile phosphate-buffered saline (PBS) (3 U/kg body weight) in the trachea of pathogen-free, eight to ten week old, female C57BL/6 N mice (n = 23, Charles River Laboratories, Sulzfeld, Germany)^21^, whereas control mice (n = 12) received 80 μl sterile phosphate-buffered saline. All animals were sacrificed 14 days^21^ after application of bleomycin or PBS and scanned immediately before the incidence. By using intraperitoneal injection of fentanyl (50 μg/kg body weight), midazolam (5 mg/kg body weight) and medetomidine (500 μg/kg body weight), mice were anesthetized for PBS/bleomycin application as well as image acquisition.

Post mortem lungs were filled with paraformaldehyde. After surgical extraction, the organs were placed into falcon tubes with paraformaldehyde for further processing.

### Imaging Protocol

All animals were scanned in a prototype small-animal scanner^22,23^ that was equipped with a built-in fan to prevent the animals from cooling down. Furthermore, the mice’s respiratory frequency and body temperature were monitored while images were acquired. The anesthetized and freely breathing mice were placed in a supine position on the sample bed in the scanner, which was operated with five stepping positions (three seconds exposure time per stepping) of the source grating. The source ran at 35 kVp and 18 W. The field-of-view was round with a diameter of 5 cm and the spatial resolution in the sample plane was 60 μm. The scanner’s grating interferometer consists of three gratings: a gold source grating (period (p) = 10 μm, height (h) = 35 μm), a nickel phase grating (p = 3.24 μm, h = 4 μm; distance to source grating 30 cm) and a gold analyzer grating (p = 4.8 μm, h = 45 μm; distance to source grating 45 cm).

### Histology

Upon washing the lungs in order to remove the paraformaldehyde, they were decalcified in 10% EDTA for five days. Afterwards, the organs were dehydrated and embedded in paraffin. At intervals of 0.5 mm multiple 10 μm slices were cut in the coronal plane. These sections were deparaffinized, hydrated, stained with Elastica van Gieson (EvG) and dehydrated again. Subsequently all slices were scanned at different magnification (MIRAX SCAN, Zeiss, Göttingen, Germany) to create digital images.

### Quantitative Morphometry

The histological slices of the lungs were analyzed with design-based stereology using an Olympus BX51 (Olympus, Germany) light microscope equipped with a computer-assisted stereologic toolbox (newCAST, Visiopharm, Hoersholm, Denmark). Slices of pulmonary tissue that covered the whole cross-section of the right and the left lung in coronal plane were evaluated. Within one of these slices 35 randomly selected regions of interest were analyzed by using a computer-aided system following published guidelines for tissue-quantification^24^. Tissue percentage, a marker for severity of fibrosis, was assessed by counting points hitting lung parenchymal tissue (P_tissue_) or lung parenchymal air (P_air_). The percentage volume was calculated by applying the formula

$$\mathrm{Tissue}- \% =\sum {{\rm{P}}}_{{\rm{tissue}}}\times 100/\sum {{\rm{P}}}_{{\rm{tissue}}}+\sum {{\rm{P}}}_{{\rm{air}}}.$$

### Quantitative Signal Analysis

In order to perform quantitative signal analysis, overlaying osseous structures (such as ribs and spine) were excluded. Signal intensities were then quantified using pentagonally shaped regions of interest (ROIs) that were placed manually into the images so that as much lung tissue as possible was captured, excluding the shadows of the mediastinum and diaphragm. The lungs of 5 animals in the control group and 10 animals in the group with fibrosis have been analyzed in a previous study using a distinct quantification algorithm^18^.

### Reader Study

Lungs from a total of 28 mice (12 control mice, 16 mice in the fibrosis group) were analyzed. Each transmission image was assessed in isolation for the presence or absence of fibrosis by two blinded, independent and experienced radiologists, familiar with dark-field imaging. Additionally, readers rated their grade of diagnostic confidence using a scale ranging from 0 (not confident), to 1 (moderately confident), 2 (quite confident) and 3 (highly confident)^25^. After an interval of two weeks both radiologists assessed dark-field images for fibrosis in the same way, and another two weeks later, corresponding transmission and dark-field images were jointly analyzed for the presence of fibrosis. The two weeks intervals were enforced to minimize recall bias. As all images were handed over as Dicom files, the readers were free to adjust the window settings according to their preference.

### Statistical Analysis

Diagnostic accuracy of readers’ visual interpretation of (a) transmission images alone, (b) dark-field images alone and (c) transmission and dark-field images combined was calculated using histopathological proof of fibrosis development as the reference standard. To test for differences in diagnostic accuracy, the DeLong method was used^26^ whereas inter-reader agreement between the observers for the assessment of fibrosis was determined using Cohen’s Kappa coefficient. Levels of agreement on κ values were defined as follows: a κ value of less than 0.2, poor agreement; a κ value of 0.21**–**0.40, fair agreement; a κ value of 0.41**–**0.60, moderate agreement; a κ value of 0.61**–**0.80, good agreement; and a κ value of 0.81**–**1.00, very good agreement^27^. The diagnostic confidence ratings were compared between the three rounds of reading using t-test. MedCalc^®^ (version 15.2.2, Ostend, Belgium) was used as statistical software.
